# Adjuvant convection-enhanced delivery for the treatment of brain tumors

**DOI:** 10.1007/s11060-023-04552-8

**Published:** 2024-01-23

**Authors:** Daniel Kreatsoulas, Mark Damante, Santino Cua, Russell R. Lonser

**Affiliations:** Department of Neurological Surgery, The Ohio State University Wexner Medical Center, The Ohio State University, N1019 Doan Hall, 410 W 10Th Avenue, Columbus, OH 43210 USA

**Keywords:** Adjuvant, Convection-enhanced delivery, Glioma, Glioblastoma, Treatment

## Abstract

**Background:**

Malignant gliomas are a therapeutic challenge and remain nearly uniformly fatal. While new targeted chemotherapeutic agentsagainst malignant glioma have been developed in vitro, these putative therapeutics have not been translated into successful clinical treatments. The lack of clinical effectiveness can be the result of ineffective biologic strategies, heterogeneous tumor targets and/or the result of poortherapeutic distribution to malignant glioma cells using conventional nervous system delivery modalities (intravascular, cerebrospinal fluid and/orpolymer implantation), and/or ineffective biologic strategies.

**Methods:**

The authors performed a review of the literature for the terms “convection enhanced delivery”, “glioblastoma”, and “glioma”. Selectclinical trials were summarized based on their various biological mechanisms and technological innovation, focusing on more recently publisheddata when possible.

**Results:**

We describe the properties, features and landmark clinical trials associated with convection-enhanced delivery for malignant gliomas.We also discuss future trends that will be vital to CED innovation and improvement.

**Conclusion:**

Efficacy of CED for malignant glioma to date has been mixed, but improvements in technology and therapeutic agents arepromising.

## Introduction

Malignant gliomas, including glioblastoma (GBM), are the most common primary brain tumors (incidence, 3 per 100,000) [1]. Malignant gliomas are heterogeneous neoplasms that are associated with wide genetic variation [2, 3]. GBM is the deadliest malignant glioma with a median survival of 16 to 20 months using current therapeutic paradigms [1, 4, 5]. To improve GBM treatment and patient prognosis, researchers have investigated the development of new targeted therapeutic compounds to treat GBM based on emerging molecular biologic understanding. Despite the enhanced effectiveness of new chemotherapeutic agents against malignant glioma in vitro, they have not been translated into successful clinical treatments. One reason for the lack of clinical effectiveness is ineffectual distribution of putative therapeutics to malignant glioma cells using conventional nervous system delivery modalities, including intravascular infusion, cerebrospinal fluid delivery and implanted polymers [6–10].

Intravascular administration of chemotherapeutic agents to malignant glioma is limited by the blood–brain barrier, heterogeneous dispersion, systemic toxicity, and non-targeted distribution. Delivery in cerebrospinal fluid (intraventricular or intrathecal) is limited by the blood-ependymal barrier, non-targeted distribution, and poor tissue penetration (2 to 3 mm). Chemotherapy-impregnated polymers placed on the walls of the tumor resection cavity have also been used to deliver anti-neoplastic agents to surrounding tissue. Because polymer drug delivery relies on diffusion-driven distribution, it is limited by poor tissue penetration (1 to 2 mm from polymer surface) that may underlie the limited effectiveness of this modality. To overcome these limitations, investigators have investigated the use of direct intraparenchymal convection-enhanced delivery (CED) to deliver putative therapeutic agents to malignant gliomas and/or the tissue surrounding resection cavities.

Convective distribution of drugs in the nervous system is driven by bulk flow of infusate via a cannula and hydraulic syringe pump. Because CED is not diffusion-driven and drug is delivered directly into the interstitial spaces of the nervous system, it is associated with unique distribution properties. Specifically, it can be used to perfuse brain tissue behind the blood–brain barrier, in a safe, targeted, reliable and homogeneous manner [11–13]. Real-time magnetic resonance (MR)-imaging can be performed to monitor infusate brain perfusion [14–16]. Based on its delivery properties, researchers have used CED to deliver putative anti-glioma agents in vitro and in clinical trials.

## Convection-enhanced delivery

### Convective delivery properties

Convective delivery relies on bulk flow distribution (driven by a small hydrostatic pressure gradient) of infusate in the nervous system interstitial spaces [11, 13]. Convective delivery systems are made from non-compliant materials that include a syringe pump and an infusate-filled syringe that is connected to an infusate cannula via infusate-filled tubing. The infusate cannula, in turn, is placed to the perfusion target (e.g. tumor bulk or surrounding brain) using direct visualization and/or stereotaxis (frame-based or frameless) [16, 17]. Because CED is not dependent on diffusion for drug distribution, it can be used to rapidly distribute small and large molecules at similar speed across the blood–brain barrier in clinically-relevant volumes [13].

### Bypasses the blood–brain barrier

Because the infusion cannula is placed within the tissue of the brain, convective delivery bypasses the blood–brain barrier. Therapeutic agents that do not cross the blood–brain barrier (i.e., highly hydrophilic compounds and/or large macromolecules) are sequestered on the abluminal side of this biological barrier. These are ideal agents for perfusion using CED [12, 13], because they can provide therapeutic exposure in the perfused region for prolonged periods of time. Alternatively, compounds that are permeable to the blood–brain barrier will rapidly transition from the interstitial space into the systemic circulation [13].

### Homogeneous distribution

Unlike diffusion-driven delivery mechanisms, the properties of bulk flow create a homogeneous distribution *(‘square-shaped’*) of therapeutic molecules across the region of perfusion [11, 12, 18]. This distribution pattern results in a uniform, high concentration of the infusate in the perfused region. The ability to achieve homogeneous concentrations of therapeutic molecules leads to a predictable and targeted pharmacologic effect in the perfused region [11, 12].

### Reliable

Because CED relies on bulk flow of putative therapeutic in the interstitial spaces, the volume of distribution (Vd) is inversely proportional to volume of infusion (Vi). The Vd:Vi ratio in normal brain (cerebrum) tissue ranges from 4 to 5:1. The Vd:Vi ratio is higher in the brainstem where the fibers are more tightly compacted and the interstitial spaces are more crowded (Vd:Vi ratio, 6 to 10:1) [11, 18]. However, factors that expand the interstitial spaces, including vasogenic edema, can reduce the Vd:Vi ratio.

### Safe

Convective delivery across a wide variety of therapeutic compounds has been performed safely in patients [19, 20]. Because normal intracranial pressures are present throughout convective delivery infusions, large volumes of infusate can be used without permanent neurological deficits. Most often, neurological symptoms immediately after CED have been transient and have been associated with local edema from perfusion of the extracellular space. These symptomatic changes can be attenuated with the administration of high-dose steroids and/or infusion stoppage [13]. Lasting neurologic findings after infusion have been secondary to local toxicity of the therapeutic agent.

### Targeted

**Fig. 1:**
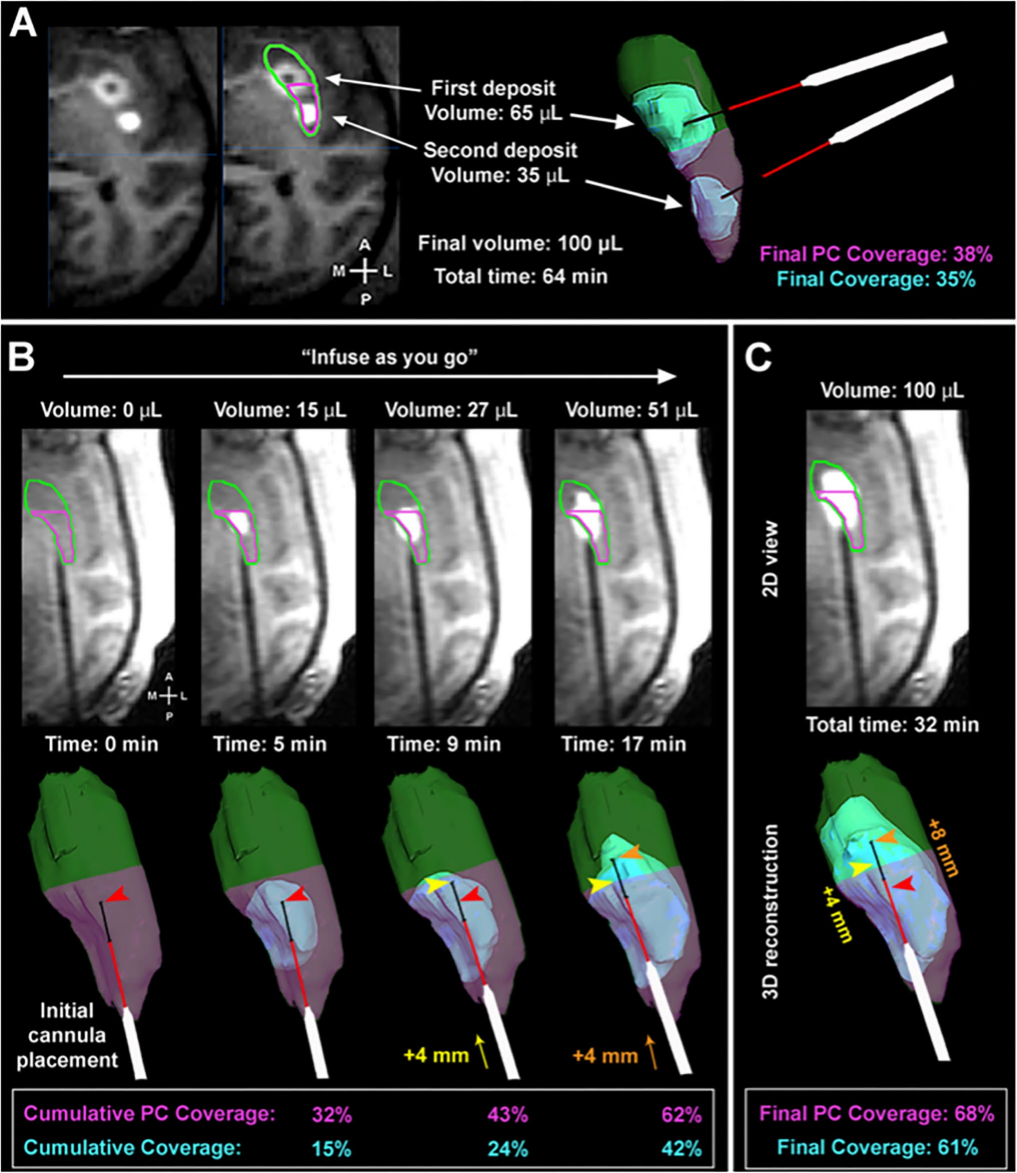
Case depiction and artist’s rendition (**A**) of a convective infusion via stereotactically placed cannula. Infusion of drug mixed with Gd-DTPA allows for tracking of distribution. (**B**-**E**) Real-time, serially obtained coronal MR images with hypodense tumor region in the pons (arrows), showing growth of the perfused area, with total tumor coverage obtained in (**E**). Figure adapted from Lonser et al. 2007 [16]

**Fig. 2:**
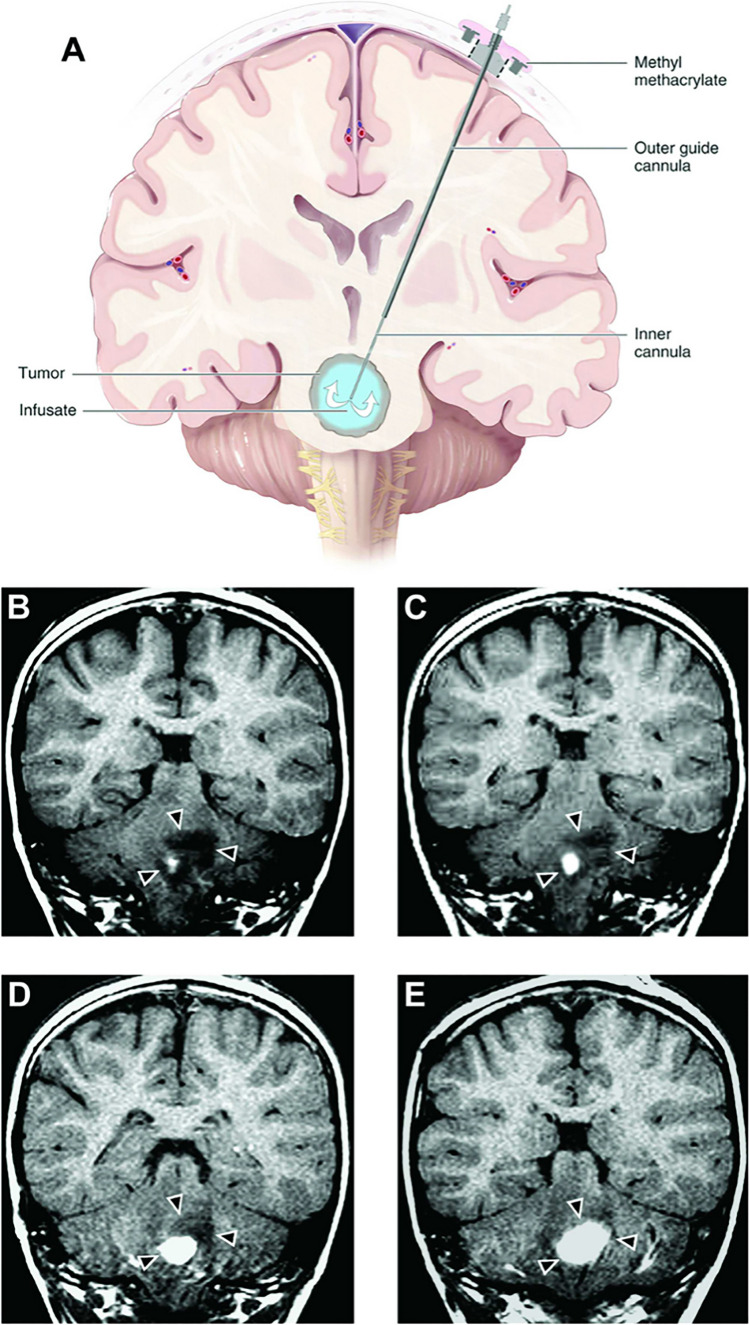
Evaluation of infusions without vs with active catheter adjustments during CED to a non-human primate putamen. (**A**) Axial MR images depicting the entire putamen (green) and the postcommissural sub-region (pink) following two separate deposits of infusate. Infusion volumes are listed in the figure. Final 3-dimensional computation of volume showed 35% final coverage achieved. (**B**) A single occipital trajectory is shown via MR images, utilizing the infuse-as-you-go method of infusion (sequential images taken, ordered left to right). Both MR image depictions (upper: whole putamen [green] and postcommissural putamen [pink]) and 3D reconstruction views (lower) are shown. The cannula (black line) is advanced in a stepwise fashion after defined volumes of infusate were delivered (100 mL total over 32 min). Improved infusion volume and target coverage can be seen over serial infusions. The cannula tip location is indicated by the colored arrowheads. (**C**) Axial MR and 3-dimensional reconstruction images depicting final putaminal coverage. The final total was 61% of putamen with 68% of the postcommisural putamen, improved coverage compared to two separate infusions. Figure adapted from Sudhakar et al. 2020 [14]

CED provides targeted delivery of therapeutics. The infusion cannula tip can be placed with high fidelity in the region targeted for perfusion including brainstem and deep nuclei targets [15–17, 21] (Fig. 1). During perfusion, infusion rates and cannula movement can be used to shape perfusion distribution and mitigate leakback (Fig. 2) [14]. This allows for the tailored perfusion of anatomic regions of the brain and tumor according to trial parameters.

### Real-time imaging of perfusion

Real-time MR-imaging of perfusion of therapeutic infusate via co-infusion of gadolinium permits the accurate tracking of pharmacologic agent distribution [15, 16, 22]. Active monitoring of the distribution of the drug permits confirmation of cannula placement, elimination of leak back, verification of adequate perfusion and improved safety through limiting perfusion to only treatment area. Using real-time MR-imaging during perfusion allows for adjustments in infusion rate and cannula position to optimally infuse the target structure/region [14]. Confirmation of target perfusion is vital to understanding the efficacy of the infused agent and safety [23].

## CED in clinical trials

Convective delivery has been utilized in clinical trials to treat either newly diagnosed or recurrent malignant glioma patients by either direct infusion into tumor bulk and/or by infusion into the tissues surrounding the tumor resection cavity. Overall, clinical trials using CED can be more complex as it is difficult (likely impossible) to have a blinded arm in randomization. CED trials for the adjuvant treatment of malignant glioma are summarized in Table 1, with ongoing trials listed in Table 2. We summarize selected trials that demonstrate the various biologic paradigms that can be used with convective delivery for malignant glioma.

**Table 1:** Completed Trials of CED for Adjuvant Therapy of Brain Tumors. Abbreviations: AA = anaplastic astrocytoma; hrBMP4 = human recombinant bone morphogenic protein-4; GBM = glioblastoma; AO = anaplastic oligodendroglioma; Gr = grade; MRI: magnetic resonance imaging; TGF-β2 = transforming growth factor beta-2; Vd = volume of distribution; WHO = World Health Organization

Author	Year	Agent	Trial phase	WHO grade, tumor type (no. of pts)	No. of catheters	Catheter location (intratumoral vs. peritumoral)	Volume infused (ml)	Mean Vd	Duration	Median OS	Adverse Events
Laske et al	1997	TF-CRM107	I	Recurrent Gr IV GBM (10), Gr III AA (5), AO (1), metastatic lung Ca (2)	1–3	Intratumoral	5–180	na	2–16 days	41 weeks (GBM patients only)	Cerebral edema, seizures
Rand et al	2000	NBI-3001 (IL4-*Pseudomonas* exotoxin)	I	Recurrent Gr IV GBM (9)	1–3	Intratumoral	30–185	na	4–8 days		
Weaver et al	2003	TF-CRM107	II	Recurrent Gr IV GBM & Gr III AA (44)	1–2	Intratumoral	40	na	4–5 days	37 weeks	Cerebral edema, seizures
Weber et al	2003	NBI-3001 (IL4-Pseudomonas exotoxin)	I	Recurrent Gr IV GBM (25) & Gr III AA (6)	1–3	Intratumoral	40–100	na	4 days	23.2 weeks (GBM patients only)	Seizures, headache, weakness, edema
Voges et al	2003	HSV-tk	I/II	Recurrent Gr IV GBM (8)	1–2	Intratumoral	30–60	3.0 (0.5–6.9) ml	29 days	28.1 weeks	Transient worsening of neurological condition, elevated body temperature
Lidar et al	2004	Paclitaxel	I/II	Recurrent Gr IV GBM (13) & Gr III AA (2)	1	Intratumoral	6	na	2–5 days	16 weeks (GBM); 30 weeks including anaplastic astrocytoma	Chemical meningitis
Patel et al	2005	Cotara*	I/II	Recurrent Gr IV GBM (37) & Gr III AA (6), newly diagnosed Gr IV GBM (8)	1–2	Intratumoral	4.5–18	na	1–2 days	37.9 weeks	Cerebral edema, hemiparesis, headache
Carpentier et al	2006	CpG oligonucleotide	I	Recurrent Gr IV GBM (24)	1–2	Intratumoral	1 ml	na	6 h	29 weeks	Lymphopenia, worsening of neurologic condition
Vogelbaum et al	2007	IL-13 PE38QQR (Cintredekin besudotox)	I	Newly diagnosed Gr IV GBM (21) & Grade III AO (1)	2–4	Peri-resection cavity	72	na	4 days	Survival range 5–113 weeks	Seizure, aphasia, confusion, fatigue, gait disturbance, nystagmus
Kunwar et al	2007	IL-13 PE38QQR (Cintredekin besudotox)	I	Recurrent Gr IV GBM (46) & Gr III AA (3) & Gr III AO (2)	1–3	Peri-resection cavity	72	17.9 cm3 ± 12.5 cm3	4 days	42.7 weeks (GBM only); 45.9 weeks all patients	Hemiparesis, convulsions, headache
Tanner et al	2007	Paclitaxel	I/II	Recurrent Gr IV GBM (8)	1–2	Intra- and Peri-tumoral	26 ml	12.8–22.9 cm^3	120 h	survival range 4–15 months	Cerebral edema, neurological deterioration, skin necrosis
Hau et al	2007	AP 12009/Trabedersen (TGF-B2 inhibitor)	I/II	Recurrent Gr IV GBM (19) & Gr III AA (5)	1	intratumoral	23–80.6 ml	na	4 days, 7 days	44 weeks (GBM)	Brain edema
Kunwar et al	2010	IL-13 PE38QQR (Cintredekin besudotox)	III	Recurrent Gr IV GBM (296)	2–4	Peri-resection cavity	72	na	4 days	45.3 weeks	Pulmonary embolism
Carpentier et al	2010	CpG oligonucleotide	II	Recurrent Gr IV GBM (34)	2	Intratumoral	2 ml	na	6 h	28 weeks	Transient neurological worsening, fever, hemorrhage at catheter site SAEs- infection, pneumoencephaly, hemorrhage
Bogdahn et al	2011	AP 12009/Trabedersen (TGF-B2 inhibitor)	IIB	Recurrent Gr IV GBM (103) & Gr III AA (42)	1	Intratumoral	40	na	7 days	36.4 weeks (GBM only)	Meningitis, hyponatremia, cerebral edema, thrombocytopenia
Bruce et al	2011	Topotecan	IB	Recurrent Gr IV GBM (10) & Gr III glioma (6)	2	Intra- and Peri-tumoral	40–100	na	4.17 days	58.5 weeks (GBM only)	Parietal syndrome, dysmetria, hemineglect, weakness
Kicielinski et al	2014	Reovirus	I	Recurrent Gr IV GBM (12) & AA (3)	1–4	Intratumoral	na	na	72 h	survival 140 days	Catheter clogging, convulsions
Desjardins et al	2018	PVSRIPO	I	Recurrent Gr IV GBM (61)	1	Intratumoral	3.25 ml	na	6.5 h	50 weeks	Hemorrhage, headache, hemiparesis, seizure
Sampson et al	2023	MDNA55 (IL4-PE	IIb	Recurrent Gr IV GBM (47)	1–4	Intra and Peri-tumoral	Up to 66 mL	Median tumor area coverage = 52.66%	< 48 h	11.6 months	Seizure, fatigue, headaches, muscular weakness
Bos et al	2023	rhBMP4	I	Recurrent Gr IV GBM (15)	3	Intra and Peri-tumoral	Up to 66 mL	27.5–125.5 cm^3	4–6 days	7.0 months	Headache, vomiting, hemiparesis, hyperglycemia

**Table 2:** Ongoing trials of CED in Adjuvant Treatment of Brain Tumors. Abbreviations: CAR T, chimeric antigen receptor T; CED, convection-enhanced delivery; GBM, glioblastoma; SPECT, single-positron emission computed tomography

Study	Tracer/Imaging Agent	Time Frame of Imaging	Trial Number	Phase	Status
D2C7 for recurrent malignant glioma	N/A	N/A	NCT02303678	1/2	Active, Not Recruiting
D2C7-IT with atezolizumab for recurrent gliomas	N/A	N/A	NCT04160494	1	Active, Not Recruiting
PVSRIPO for recurrent GBM	N/A	N/A	NCT01491893	2	Completed
PVSRIPO and pembrolizumab in patients with recurrent glioblastoma (LUMINOS-101)	N/A	N/A	NCT04479241	2	Active, Not Recruiting
EGFRvIII CAR T cells for recurrent GBM (INTERCEPT)	111-Indium-labeled CAR-T cells (SPECT-traceable)	2 days post-infusion	NCT03283631	1	Closed
186Rhenium nanoliposomes (186RNL) in recurrent glioma	186-Rhenium-labeled liposomes (SPECT-traceable)	Up to 7 days post-infusion	NCT01906385	1/2	Recruiting
Topotecan via CED for recurrent grade III/IV glioma	Gadolinium-based agent	Real-time	NCT03927274	1	Terminated (Lack of funding)
Nanoliposomal irinotecan for recurrent high-grade glioma	Gadolinium-based agent	Within 2 days post-infusion	NCT02022644	1	Completed
Convection-enhanced delivery of OS2966 for patients with high-grade glioma undergoing a surgical resection	Gadoteridol	Within 1 day post-infusion	NCT04608812	1	Terminated (Enrollment)
Safety & Efficacy/​Tolerability of Rhenium-186 NanoLiposomes (186RNL) for Patients Who Received a Prior 186RNL Treatment	186-Rhenium-labeled liposomes (SPECT-traceable)	3 days post-infusion	NCT05460507	1	Not yet recruiting
D2C7-IT in Combination With 2141-V11 for Recurrent Malignant Glioma	N/A	N/A	NCT04547777	1	Recruiting

### Conjugated immunotoxins

#### Transferrin-receptor ligand conjugate

The transferrin receptor is highly expressed on malignant gliomas. A transferrin receptor ligand-*Diphtheria* toxin B subunit conjugate (Tf-CRM107) [24] was used to treat 18 patients (Phase I) with recurrent histologically proven GBM [25]. Fifteen patients were available for analysis and 9 patients demonstrated a 50% or greater reduction in tumor volume at follow-up. Patients tolerated the procedure well and median survival in patients with a radiographic response was 74 weeks, compared to 36 weeks in the non-responder group. A Phase II trial was performed that included 44 patients [24]. Thirty-four patients were eligible for efficacy and survival analysis. Thirty-nine percent of patients responded to treatment (20% partial responses and 15% complete responses) and 30% of patients survived longer than 1 year from treatment. A Phase III trial was initiated but was closed because of a low objective response rate at interim reporting [26].

#### Interleukin-4-Pseudomonas exotoxin (IL4-PE)

Interleukin-4 (IL4)-Pseudomonas exotoxin conjugate (IL4-PE) targets glioma cells by binding the IL4 receptor, which is highly expressed by malignant gliomas [27]. A Phase I trial included 31 patients with recurrent malignant glioma (25 GBM) that underwent CED infusions of IL4-PE in a dose-escalating manner [28].This trial showed no systemic toxicity and adverse events were mostly mild and limited to the CNS. However, improvement in survival was not significant (median survival after infusion: 5.8 months).

A second formulation of IL4-PE (MDNA55) that is more specific to IL4 receptor-expressing cells was created for CED. A Phase IIb trial of this form of IL4-PE delivered via CED to 47 patients with at GBM recurrence [29]. To better understand the treatment population, IL4 receptor expression in each tumor was evaluated in this trial and distribution was assessed immediately postoperatively via gadolinium co-infusion. Median overall survival for the intention-to-treat and as-treated groups were 10.2 months and 11.6 months. Median tumor coverage was 53%. Failure to extend survival may have been secondary to poor coverage of tumor. However, survival was lengthened to 15.0 months in patients with high IL4R expression receiving any dose, as well as low-expression IL4R patients receiving high doses.

#### Cintredekin Besudotox

Cintredekin besudotox (IL13-PE38QQR) is a recombinant protein which binds to the IL-13 receptor and acts as a highly specific immunotoxin against glioma cells [30, 31]. Two Phase I trials of IL13-PE38QQR treated 51 (46 GBM) and 22 (21 GBM) patients malignant glioma patient. Both trials reported improved survival outcomes (median survival 45.9 weeks and 44.0 weeks, respectively). These encouraging data led to a Phase III trial.

A blinded randomized Phase III study compared IL13-PE38QQR to drug-impregnated polymer (Gliadel) placement in GBM patients after resection of first recurrence [23]. Two hundred-ninety six patients (188 evaluable for efficacy analysis) were randomized to receive IL13-PE38QQR or drug-impregnated polymer (2:1 ratio). There was no median significant survival difference between groups (36.4 weeks for IL13-PE38QQR-treated patients compared to 35.3 weeks for the polymer-treated patients). However, tumors were not assessed for IL13 receptor expression, which may confound patient response to IL13-PE38QQR therapy. Moreover, only 68% of catheters were appropriately positioned.

### Oncolytic Virus

#### HSV-tk Gene

The herpes simplex virus-1 carrying the thymidine kinase gene (HSV-tk) has been used to treat high grade glioma via infection of cells and sensitization to treatment with antiviral therapy [32–34]. Voges and colleagues treated 8 patients with unresectable, progressive malignant glioma via CED of HSV-1-tk gene liposomal complex and systemic ganciclovir after a preoperative “test” infusion of gadolinium as a surrogate for drug distribution. The test infusions demonstrated a Vd:Vi ratio of 3.0 (range, 0.5 to 6.9) and distribution heterogeneity within tumors and between patients. While colocalization of anti-tumor activity with vector gene expression was found, there was not a reported survival benefit.

#### Poliovirus Sabin rhinovirus (PVSRIPO)

PVSRIPO is a live attenuated, recombinant poliovirus type 1 that enters tumor cells with high specificity and killing efficacy via activation of native antiviral immune responses. A Phase I trial of this modified poliovirus delivered via CED was performed in 61 patients with recurrent GBM [35]. Trial patients were compared with historical controls from the same center. The infusions were well-tolerated. There was no significant improvement in survival (patients receiving treatment demonstrated median survival of 12.5 months versus 11.3 months for historical controls). Twenty-one percent of treated patients were alive at 36-month follow up compared to 4% of controls.

### Traditional Chemotherapeutics

#### Paclitaxel

Paclitaxel is a chemotherapy agent used in conventional chemotherapy regimens that is shown to be active against malignant glioma in animal models when delivered by CED [36, 37]. Fifteen patients (13 GBM, 2 anaplastic astrocytoma) were evaluated in a Phase I/II study delivering 20 cycles of intratumoral paclitaxel via CED [38]. There was an overall response rate of 73% (5 complete responses, 6 partial responses). Histologic evaluation of resected tissue from 5 patients showed widespread necrosis and early gliotic changes. Despite these indicators of response, no significant improvement in survival was seen.

#### Topotecan

High grade gliomas have been shown to express topoisomerase I at a higher rate than other cancers [39, 40]. Agents that target this enzyme (e.g., topotecan) are potent cytotoxic agents but have failed to produce results when given systemically in malignant glioma. A Phase I trial evaluated topotecan delivered by CED, in 16 high-grade glioma patients, performing CED after resection/biopsy was performed [41]. Overall, 25% of patients were alive 105 weeks or more after infusion, with median survival of greater than 1 year. Seven patients (46%) had disease stability for 6 months or more. Topotecan is actively being studied with a multiport catheter system [42] and via chronic CED [43].

#### Carboplatin

Carboplatin is a platinated chemotherapeutic agent which covalently binds DNA, leading to DNA damage and cellular apoptosis after treatment. It has been utilized systemically as a treatment for malignant glioma with little efficacy [44, 45]. Wang and colleagues evaluated escalating doses of carboplatin, infused over 72 h via CED. Ten patients (Phase I) with recurrent HGG were treated [46]. Overall, survival outcomes were not improved (median survival was under 10 months) and there was no significant improvement in progression-free survival.

### Other Agents

#### CpG Oligonucleotides

An emerging technology to treat solid cancers has been oligonucleotides containing CpG motifs, which strongly activate an immune response when injected locally [47, 48]. A Phase I trial enrolled 24 patients to receive escalating CED doses of CpG-oligodeoxynucleotide-28 (CpG-28) [49]. Patients in this trial experienced many adverse events (120 total, 63 infusion-related), including lymphopenia (n = 36). Median survival (7.2 months) was not extended but overall survival at 1 year was reported at 28%. These findings led to a Phase II trial [50] where 31 patients were treated with CpG-28. Adverse events, inlcuding lymphopenia, were again common throughout the patient cohort. However, improvements in progression-free survival and overall survival were not reported.

#### Trabedersen

Transforming growth factor-beta (TGFb) is a molecule known to lead to glioma progression, immune evasion, and mesenchymal transition [51–53]. Subsequently, it has been targeted with a drug called AP-12009 (trabedersen) [54]. Trabedersen was used in 3 separate phase I/II studies (reported in aggregate) via subcutaneous pump placement attached to a catheter to perform CED [55]. Infusions were well-tolerated with minimal systemic adverse events. Overall, there was no significant improvement in survival, but when stratified by tumor histology, anaplastic astrocytoma patients had significantly improved median survival (146.6 weeks) while GBM patients had minimal improvement (44.0 weeks). Two anaplastic astrocytoma patients had long-term remissions with recurrence at delayed time points (10 and 22 months after infusions).

A randomized Phase IIb trial used CED infusion of AP-12009 in 2 different concentrations and outcomes were compared with standard chemotherapy in 145 recurrent high grade glioma patients (103 GBM, 42 anaplastic astrocytoma) [54]. Survival was similar among two different concentrations and the standard chemotherapy group. When categorized histologically, anaplastic astrocytoma patients again did not significantly increase survival on AP-12009 compared to traditional chemotherapy. Their median overall survival was 39.1 months (10 uM formulation) and 35.2 months (80 uM) for AP-12009 versus 21.7 months for standard chemotherapy. Overall, improvement in survival was not seen in GBM patients. Importantly, it was found that standard early-disease trial endpoints (6 month or 1 year survival) may not be appropriate for immune-based therapies, as the patients that did respond to treatment only became apparent at the 14-month evaluations.

#### Human Recombinant Bone Morphogenic Protein-4

Human recombinant bone morphogenic protein-4 (hrBMP4) treatment has been shown to reduce glioma stem cell populations via reduction of stemness and pro-differentiation effects [56]. hrBMP4 (Phase I) was infused via CED in 15 patients with recurrent malignant glioma. Distribution was assessed with MR-imaging at 24 h after beginning and at the end of infusion. Mean Vd:Vi ratio was 0.7 (range, 0.3 to1.1). Mean tumor coverage of 16% (range, 4 to 39%). Progression free and overall survival were not improved. However, radiographic responses were seen and recurrences were rare in areas where rhBMP4 had been infused [56].

## Future Directions

A combination of factors, including lack of drug efficacy, and suboptimal distribution of therapeutic agent, may have led to failures to improve outcomes for malignant glioma patients with CED. To overcome these issues, current work is focused on developing improved putative therapeutic agents, real-time imaging during infusion, infusion hardware improvements and delivery improvements.

### Putative therapeutic development

Emerging therapeutics are exploiting recent advances in understanding of high-grade glioma genetics and pathobiology. These therapeutics are often tailored to specific subsets of malignant gliomas through genetic expression analysis and targeting specific tumor antigen expression. Specifically, ongoing in vitro research and clinical trials are exploring directed infusion of targeted immunotoxins, viral vectors, gene therapy and immune-based treatment (Table 2).

### Real-time MR-imaging

Real-time MR-imaging can be used to ensure accurate infusion cannula placement and to define the region of perfusion (therapeutic coverage). Imaging can also be used to shape infusions in real-time and ameliorate infusate leak back along the infusion cannula. [14, 16, 17, 22, 23, 30, 58, 59]. Ultimately, real-time MR-imaging will permit better understanding of convective infusion parameters and properties in tumor and the surrounding regions of brain. It will also allow for defined technical assessment (e.g., coverage of desired targets/anatomic regions) and better inform efficacy (or lack thereof).

### Infusion hardware improvements

Several infusion hardware developments will enhance CED for malignant glioma in the future. Specifically, improved cannula design (e.g., multiport catheters), improved MR-imaging compatible frameless stereotactic systems (e.g., ball joint guide array) and robotics for cannula placement are being explored to improve the efficiency and effectiveness of convective delivery for malignant gliomas and other neurologic disorders. Multiport catheters and models that mimic brain porosity have been developed to improve degree and selectivity of perfusion along the catheter [42, 60]. New targeting technologies, including the ball-joint guide array allow for rapid placement of multiple cannula tracts through a single burr hole [17], and cranial robotics platforms can be used to place catheters rapidly with high accuracy [61, 62].

### Clinical delivery improvements

Developments in convective delivery are being studied to enhance distribution to include long-term outpatient delivery (i.e.,). A recent Phase I trial used chronic, metronomic CED infusions of topotecan via a subcutaneously implanted catheter and pump (5 patients, 48-h delivery with 5–7 day washouts between cycles) [43]. The catheters were placed in recurrent tumor and, after infusion cessation, patients underwent resection of the tumor. Pre- and post-treatment tissue analysis and showed an increase in gene signatures associated with apoptosis and DNA damage in the tumors. The infusions were well tolerated.

## Conclusion

CED for the treatment of malignant glioma has demonstrated feasibility and safety of delivering a variety of different anti-glioma agents in patients. Prior trials have not shown significant improvement in survival for several reasons, include lack of perfusion tracking/confirmation with imaging, heterogeneous/lack of glioma-associated target expression and/or poor drug distribution in heterogeneous tissue environment. Recent advances have been developed to overcome these potential limitations in future trials.

## Funding

None.

## Declarations

No institutional review board approval was required for this manuscript.
